# Addressing Gun Safety and Dementia: Training Professionals in the Community

**DOI:** 10.1093/geroni/igaa057.903

**Published:** 2020-12-16

**Authors:** Silvia Orsulic-Jeras, Sara Powers, Jessica Empeno, Regina Lagasca, Amy Abrams, Sarina Barker, Rebecca McDaniel, Marcie Hanna

**Affiliations:** 1 Benjamin Rose Institute on Aging, Cleveland, Ohio, United States; 2 Lightbridge Hospice & Palliative Care, San Diego, California, United States; 3 Alzheimer’s San Diego, San Diego, California, United States

## Abstract

As the number of Alzheimer’s disease and related dementia (ADRD) cases grow, first responders will have more frequent interactions with individuals living with ADRD within the community. Recognizing the lack of dementia training among first responders and through the support of an Administration for Community Living grant, Alzheimer’s San Diego partnered with local law enforcement to support and educate first responders on local ADRD resources, referral systems for families and caregivers, and disease-related information, in hopes of building stronger dementia capable services within San Diego County. To date, 249 first responders have participated in dementia education trainings and completed the related evaluations that aimed to understand their experiences with and knowledge of dementia. On average, participants were 44.74 years old (SD=18.45) and served 8.72 years (SD=7.99) on their job. Majority were male (n=189), had some college education (n=94), and identified as White/Caucasian (n=105) or Hispanic/Latino (n=92). Although objective knowledge (as measured by a shortened version of the Alzheimer’s Disease Knowledge Scale [ADKS]) did not significantly differ from pre- to post-test, participants’ perceived subjective knowledge about dementia significantly increased from pre-test (M=1.17, SD=0.77) to post-test (M=1.89, SD=0.66); t(141)=10.56, p<.001. Encouragingly, participants who reported receiving prior training on dementia had significantly higher ADKS scores at both pre- and post-test compared to those who did not have prior training. Results highlight the need for ongoing dementia-related training for law enforcement and the importance of evaluation requirements. Discussion will focus on policy implications and how communities can support dementia capability among first responders.

